# Lactational and geographical variation in the concentration of six oligosaccharides in Chinese breast milk: a multicenter study over 13 months postpartum

**DOI:** 10.3389/fnut.2023.1267287

**Published:** 2023-09-05

**Authors:** Shuang Liu, Yingyi Mao, Jin Wang, Fang Tian, David R. Hill, Xiaoying Xiong, Xiang Li, Yanrong Zhao, Shuo Wang

**Affiliations:** ^1^Tianjin Key Laboratory of Food Science and Health, School of Medicine, Nankai University, Tianjin, China; ^2^Abbott Nutrition Research & Development Center, Shanghai, China; ^3^Abbott Nutrition Research & Development Center, Columbus, OH, United States

**Keywords:** human milk, oligosaccharides, variation, lactation, geography

## Abstract

**Introduction:**

Understanding the variations of oligosaccharide in breast milk contribute to better study how human milk oligosaccharides (HMOs) play a role in health-promoting benefits in infants.

**Methods:**

Six abundant HMOs, 2’-fucosyllactose (2’-FL), 3-fucosyllactose (3-FL), Lacto-N-tetraose (LNT), Lacto-N-neotetraose (LNnT), 3’-sialyllactose (3’-SL) and 6’-sialyllactose (6’-SL), in breast milk collected at 0–5 days, 10–15 days, 40–45 days, 200–240 days, and 300–400 days postpartum from six locations across China were analyzed using high-performance anion-exchange chromatography-pulsed amperometric detector.

**Results:**

The concentration of individual HMO fluctuated dynamically during lactational stages. The median ranges of 2’-FL, 3-FL, LNT, LNnT, 3’-SL, and 6’-SL across the five lactational stages were 935–2865 mg/L, 206–1325 mg/L, 300–1473 mg/L, 32–317 mg/L, 106–228 mg/L, and 20–616 mg/L, respectively. The prominent variation was observed in the content of 6’-SL, which demonstrates a pattern of initial increase followed by a subsequent decrease. Among the five lactational stages, the transitional milk has the highest concentration, which was 31 times greater than the concentration in mature milk at 300–400 days postpartum, where the content is the lowest. Geographical location also influenced the content of HMOs. LNT and LNnT were the highest in mature milk of mothers from Lanzhou among the six sites at 40–240 days postpartum. Breast milks were categorized into two groups base on the abundance of 2’-FL (high and low). There was no significant difference in the proportions of high and low 2’-FL phenotypes among the six sites, and the percentages of high and low 2’-FL phenotypes were 79% and 21%, respectively, across all sites in China.

**Discussion:**

This study provided a comprehensive dataset on 6 HMOs concentrations in Chinese breast milk during the extended postpartum period across a wide geographic range and stratified by high and low 2’-FL phenotypes.

## 1. Introduction

WHO recommend exclusive breastfeeding for the first 0–6 months and continued breastfeeding up to 2 years and beyond with appropriate complementary foods (1). Breast milk is associated with a lower incidence of diarrhea, obesity and infectious disease in infants by the combined action of macronutrients, micronutrients, and bioactive components (2–5). Human milk oligosaccharides (HMOs) are the third-largest solid component of human milk after lactose and fat (6). At least 150 HMOs have been identified in human milk using advanced detection technology (4). Fucosylated HMOs represent 45–83% of total HMO content, acetylated HMOs represent 6–35% of total HMO content, and sialylated HMOs represent 6–21% of the total HMO content (7–13). 2′-fucosyllactose (2′-FL), 3-fucosyllactose (3-FL), Lacto-N-tetraose (LNT), Lacto-N-neotetraose (LNnT), 3′-sialyllactose (3′-SL), and 6′-sialyllactose (6′-SL) are representative of the three major categories of HMOs and are among the most abundant and extensively studied individual structures. An increasing number of preclinical studies revealed that HMOs have health-promoting benefits, including the selective proliferation of intestinal microbiota such as Bifidobacterium and Lactobacillus (14–17), inhibiting pathogen binding to receptors on intestinal epithelial cells by mimicking structural features of histo-blood group antigens (HBGAs) (18–20), modulating immune function by binding to pattern recognition receptors, cytokine receptors, and other types of receptors (21–23), and promoting brain and cognitive development by participating in the formation of gangliosides and polysialic acid chains in the brain through a long-term potentiation effect mediated by the vagus nerve and/or systemic distribution via the circulation (24, 25).

Multiple previous studies have indicated a wide range of HMO variation in breastmilk. For example, fucosylated HMOs in breastmilk ranges from 0.02 to 6 g/L (4). Lactational stage and maternal phenotype may contribute to variations in HMO content and composition. The HMO content is highest in colostrum, about 20–25 g/L, and tends to decrease over the course of lactation to about 5–15 g/L in mature milk (6, 9, 12, 26, 27). Maternal HMO phenotype is determined in part by two alleles of the secretor (Se) and Lewis (Le) genes. The presence of α1-2-fucosyltransferase (FUT2) in secretor-positive mothers can link fucose to the core structure through α1-2 linkage, resulting in HMOs with an α1-2 linkage structure in expressed breast milk, such as 2′-FL and Lacto-N-fucopentaose I (LNFP I). Lewis-positive mothers have α1-3-fucosyltransferase (FUT3) that can link fucose to the core structure through α1–3/4 linkage, resulting in HMOs with α1–3/4 linkage such as 3-FL and Lacto-N-fucopentaose II (LNFP II) are abundantly present in breast milk (28–30). In a study by Lefebvre et al. (31), the proportion of Se+ and Se− mothers in Germany was 88%: 12% and the proportion of Le+ and Le− mothers was 94%: 6%.

In addition to lactational stage and maternal phenotype, other evidence has shown that the concentration of HMOs varies by geographical location. An international cohort study demonstrated that the average concentration of LNT in breast milk collected in European (Spanish and Swedish) and African (Ethiopian, Ghanaian, and Kenyan) was higher than in a South American (Peruvian) population (13). Breast milk from a cohort in Spain was shown to have a higher mean concentration of 6′-SL relative to a cohort from Sweden. Even within the same country, study subjects from California expressed higher levels of Lacto-N-fucopentaose III (LNFP III) relative to a comparable study cohort from Washington (13). Geographic variation in the proportion of secretors and non-secretors has also been observed. A recent study reported that the percentage of secretor-positive mothers from South America was higher than in Africa (32). These data indicate that there are differences in HMO concentration even within the same geographical region, and together with the variation in the frequency of maternal phenotypes between populations, suggest the need for a large-scale multicenter study to determine the range and variation in HMO concentration in breast milk.

Currently, several important HMOs including 2′-FL, 3-FL, LNT, LNnT, 3′-SL and 6′-SL are safe for use in infant formula according to the European Food Safety Authority and the Food and Drug Administration, and are available in large-scale commercial production (33–36). Hence a comprehensive dataset on HMOs profile in Chinese breast milk is useful to fortify Chinese infant formula products with HMOs. Several previous studies have reported on the content of HMOs in Chinese breast milk (10, 26, 28, 37–40). The MING study reported the content of 10 HMOs in 446 breast milk samples from 0–240 days postpartum in three cities in China (10). In a study by Ma L et al., the concentration of HMOs in 140 breast milk samples over 30–240 days postpartum was reported (26). In a large cross-sectional study conducted in eight provinces of China (*n* = 6,481), Ren et al. (39). randomly analyzed 481 breast milk samples to determine the concentrations of 24 HMOs and the distribution of the Lewis phenotype. Additionally, this study investigated the relationship between various clinical data of mothers and infants and the levels of HMOs. Our previous study investigated the variation in HMO concentration in breast milk collected from one city over a prolonged breastfeeding period (37). However, to the best of our knowledge, there is limited data available on HMO concentration over a prolonged period of lactation in Chinese populations. In addition, the HMO profile of milk from mothers with different Se phenotypes has not been determined in China with a larger-size number of participants. Hence, the primary aim of this study was to conduct a comprehensive study of the abundance of six of the major HMOs, representative oligosaccharides for each of the three categories, in breast milk collected from different regions of China over a prolonged breastfeeding period. In this study, we analyzed HMOs in 2,616 breast milk samples from 1,758 healthy mothers at 0–13 months postpartum collected across six representative sites in China using high-performance anion-exchange chromatography-pulsed amperometric detector (HPAEC-PAD). This study expanded our knowledge of HMO concentration in Chinese breast milk and analyzed HMO composition in high 2′-FL and low 2′-FL phenotypic subgroups. This data is useful as a scientific basis for HMO fortification of infant formula and ongoing evaluation of HMO composition in China.

## 2. Materials and methods

### 2.1. Study design and participants

The results presented in this study are a part of the Maternal Nutrition and Infant Investigation (MUAI) study aimed to explore the relationship between nutrient levels in breast milk and infant growth outcomes. The purpose of this study was to investigate geographic variation in HMOs concentrations and the frequency of the secretor phenotype across multiple geographic locations in China. Breast milks were collected from mothers in six cities, including Northeast (Changchun), Northwest (Lanzhou), Southwest (Chengdu), East (Tianjin), South (Guangzhou), and a metropolitan city (Shanghai). Five stages of lactation were chosen to explore the content of HMOs in colostrum’s period (0–5 days postpartum), transitional milk’s period (10–15 days postpartum), mature milk’s period (40–45 days postpartum), the period during complementary food introduced (6–8 months postpartum) and one year after delivery (11–13 months postpartum). The recruitment and exclusion criteria for mother-infant pairs were consistent with our previously published literature (37). Healthy mother-infant pairs meeting the following criteria were recruited: mothers aged between 20 and 35 years old, living in the local area for more than 2 years, and single childbirth. The baby’s gestational age is between 37–42 weeks and Apgar score over 8. All subjects gave their informed consent for inclusion before they participated in this study. This study was conducted in accordance with the Declaration of Helsinki, and the protocol was approved by the Ethics Committee of Tianjin hospital of ITCWM Nankai hospital. This study was registered in the China Clinical Trial Center (ChiCTR1800015387).

### 2.2. Breast milk sample collection

The volumes of breast milk samples collected at 0–5 days, 10–15 days, 40–45 days, 200–240 days, and 300–400 days postpartum were 8–10 mL, 10–15 mL, 50–100 mL, 50–100 mL, and 50–100 mL, respectively. Samples from lactating mothers were collected by extracting total milk from one side of the breast using either a manual or an electric breast pump between 8: 00–11: 00 AM on the designated collection day. After collecting the breast milk, it was gently inverted 10 times before transferring the specified volume into a foil-wrapped tubes. The remaining milk was then returned to the lactating mother. The breast milk samples in foil-wrapped tubes were delivered to the local laboratory in each city as soon as possible at 4°C. Upon arrival at the local laboratory, the samples underwent a 20-min incubation in a water bath set at 25°C, followed by a 2-min vortexing process to ensure complete homogenization. This step ensured that the top layer of breast milk was free from any solid or semi-solid greasy components, and there were no small particle attachments on the inner surfaces of the tubes. The breast milk samples were then divided into 1.2 mL sub-packages and the resulting aliquoted samples were subsequently stored at a constant temperature of −80°C. All subpackaged samples were transported every 6 months through a cold-chain transportation at-80°C to a central laboratory in Shanghai. The analysis of HMOs was carried out at the central laboratory.

### 2.3. HMOs analysis

The determination of 6 HMOs in breast milk samples was conducted using a well-established method, employing high-performance anion exchange chromatography with a pulsed amperometric detector (HPAEC-PAD), as previously described (37). For colostrum and transitional milk samples, 0.1 milliliter of breast milk was mixed with 1.9 milliliters of laboratory water. For breast milk collected at 40–400 days postpartum, 0.2 milliliter of breast milk was mixed with 1.8 milliliters of laboratory water. Filter through a 0.22 μm nylon filter into an autosampler vial, aiming to remove a fraction of the high-molecular-weight proteins and fats to reduce the load on the chromatography column. The HPAEC-PAD analyses were performed on a Thermo Fisher HPAEC ICS 5000 series instrument (Thermo, USA), utilizing a CarboPacTM PA1 Analytical column (4 × 150 mm, Thermo) connected to a CarboPacTM PA1 Guard column (4 × 50 mm, Thermo). The separation of the 6 types of HMOs was achieved using elution gradients at a flow rate of 1.0 mL/min and a temperature of 25°C. For the separation of 2′-FL, 3-FL, LNT, and LNnT, the elution gradients were as follows: 0.00–12.00 min, 60 mM NaOH; 12.00–20.00 min, 60–155 mM NaOH; 20.00–30.00 min, 155 mM NaOH; 30.01–43.00 min, 125 mM NaOH/18 mM NaOAc; 43.01–48.00 min, 100 mM NaOH/240 mM NaOAc; 48.01–63.00 min, 60 mM NaOH. For the separation of 3′-SL and 6′-SL, the elution gradients were as follows: 0.00–19.00 min, 100 mM NaOH/60 mM NaOAc; 19.01–23.00 min, 100 mM NaOH/240 mM NaOAc; 23.01–30.00 min, 100 mM NaOH/60 mM NaOAc. The quantification of HMOs was performed using the external standard method.

### 2.4. Statistical analysis

All exploratory and descriptive statistical analyses were performed with SPSS 25.0 (SPSS, Inc., Chicago, IL, USA). The statistical analysis method was selected based on normality plots with tests and homogeneity of variance testing of the data. HMO concentrations were reported as the median (p25, p75) by Weighted Average (Definition 1) since the data presented a non-normal distribution. Statistical analyses were considered significant at *p* < 0.05 using two-sided tests. The difference in continuous variables according to maternal characteristics was evaluated by the Kruskal Wallis Test, and distributions across groups were compared by Kruskal-Wallis One-way ANOVA (multiple comparisons, all pairwise, adjusted *p* value using the Bonferroni correction for multiple tests). The difference of categorical variables in maternal characteristics was compared by Chi_square Test, and the proportions across groups were compared by z-test with adjusted *p*-values (Bonferroni method). The variation of HMO concentrations among lactational stages and geographical sites was explored with Independent Nonparametric Test (Kruskal-Wallis One-way ANOVA, all pairwise, adjusted *p* value by the Bonferroni correction for multiple tests). The proportions of secretor and non-secretor were compared by Chi-square Test.

## 3. Results

### 3.1. Basic characteristics

A total of 1,758 healthy mothers participated in the study, including 244 from Changchun, 299 from Lanzhou, 281 from Chengdu, 229 from Tianjin, 342 from Guangzhou, and 363 from Shanghai. The description of the sample size within each lactational period in each city was presented in Supplementary Figure S1. Overall, the MEAN (SD) age at delivery of the participant mothers was 29.9 (3.5) years. The pre-pregnancy BMI and pre-delivery BMI of the participant mothers was 21.1 (2.9) kg/m^2^ and 26.6 (3.3) kg/m^2^, respectively. The weight gain during pregnancy was 14.6 (5.6) kg. The gestational age of the participant mothers was 39.2 (1.6) weeks. The rate of vaginal delivery and primipara was 59 and 71%, respectively. 49% of infants were female. The mean infant birth-weight and birth-length were 3,397.6 (637.9) g and 49.9 (2.1) kg, respectively. The characteristics of participant mothers in each city are listed in Table 1. Except for the maternal age at delivery and the infant’s sex, the characteristics of mothers and infants showed significant differences across cities.

**Table 1:** Characteristics of participant mothers in each city (MEAN ± SD or %).

Characteristic	Changchun (*n* = 244)	Lanzhou (*n* = 299)	Chengdu (*n* = 281)	Tianjin (*n* = 229)	Guangzhou (*n* = 342)	Shanghai (*n* = 363)	*P* value
Maternal							
Age at delivery, years	29.9 ± 3.0	29.8 ± 4.3	29.5 ± 3.3	30.3 ± 3.2	29.7 ± 3.7	30.1 ± 3.0	0.054
Pre-pregnancy BMI, kg/m^2^	21.3 ± 3.0 ^a,b^	21.3 ± 2.8 ^a,b^	20.7 ± 2.5 ^b^	21.8 ± 3.4 ^a^	20.3 ± 2.5 ^b^	21.2 ± 2.8 ^a,b^	<0.001
Pre-delivery BMI, kg/m^2^	27.4 ± 3.8 ^a^	26.8 ± 3.0 ^a,b^	26.2 ± 2.8 ^b^	27.3 ± 3.4 ^a^	25.5 ± 3.1 ^b^	26.7 ± 3.6 ^a,b^	<0.001
Gestational weight gain, Kg	16.5 ± 5.5 ^a^	14.3 ± 5.3 ^b^	14.2 ± 4.6 ^b^	14.6 ± 7.6 ^b^	13.5 ± 4.9 ^b^	14.7 ± 5.5 ^b^	<0.001
Gestational age, weeks	39.0 ± 1.1 ^b^	39.0 ± 2.1 ^a,b^	39.5 ± 1.6 ^a^	39.2 ± 1.7 ^a,b^	38.9 ± 1.4 ^b^	39.6 ± 1.0 ^a^	<0.001
Vaginal delivery, %	42% ^a^	59% ^b^	43% ^a^	68% ^b,c^	77% ^c^	61% ^b^	<0.001
Primipara, %	95% ^a^	50% ^b^	73% ^c,d^	77% ^d^	64% ^c^	74% ^c,d^	<0.001
Infant							
Birthweight, g	3,380.1 ± 401.1 ^a,b^	3,277.8 ± 445.1 ^b^	3,409.5 ± 624.7 ^a,b^	3,396.7 ± 490.8 ^a^	3,542.6 ± 1,004.9 ^a,b^	3,332.3 ± 368.2 ^a,b^	0.025
Birth-length, cm	50.5 ± 1.7 ^a^	49.0 ± 2.5 ^c^	49.8 ± 1.5 ^b,c^	50.4 ± 2.6 ^a,b^	50.3 ± 2.3 ^b^	49.4 ± 1.3 ^c^	<0.001
Female infant, %	49%	47%	43%	51%	48%	53%	0.159

^a–d^Values within a row in individual cities with different superscript letters were significantly different (adjusted *p* < 0.05).

### 3.2. Concentrations of six HMOs over lactational stage and geographical sites

The median (p25, p75) values of six HMOs in 2,616 breast milk samples collected at 0–13 months postpartum were 2′-FL 1,466 (624, 2,410) mg/L, 3-FL 665 (259, 1,310) mg/L, LNT 555 (299, 1,072) mg/L, LNnT 97 (39, 199) mg/L, 3′-SL 125 (100, 165) mg/L, and 6′-SL 190 (29, 461) mg/L. The median (p25, p75) values of total six HMOs was 3,467 (2,879, 4,672) mg/L.

**Figure 1:**
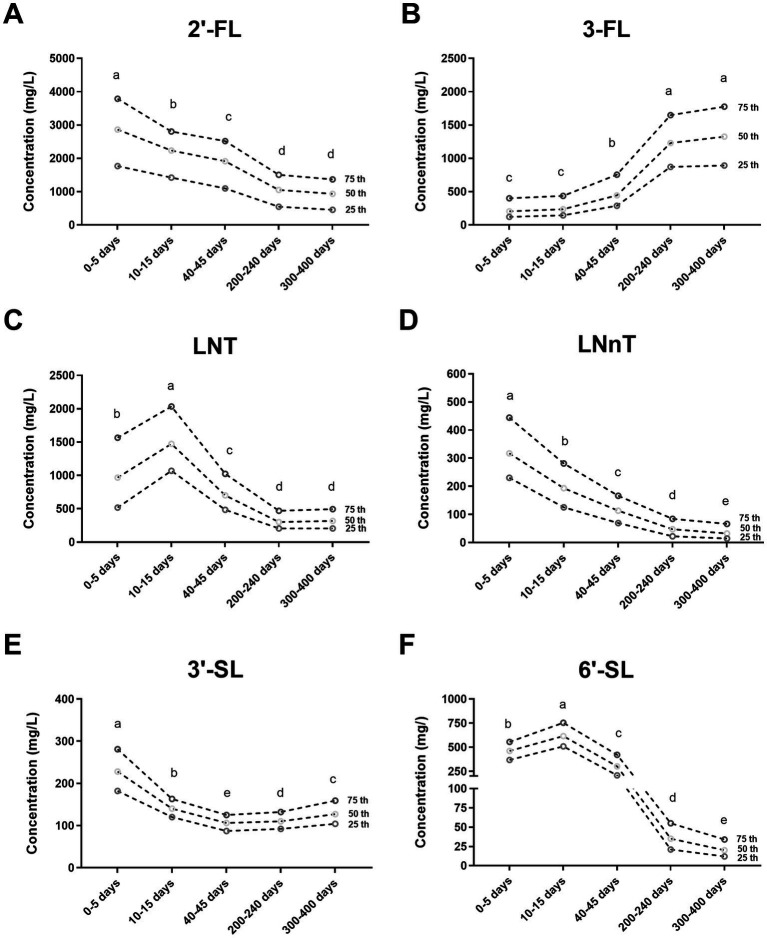
The concentration and variation of six human milk oligosaccharides (HMOs) at 0–13 months postpartum **(A–F)**. 2′-FL, 2′-fucosyllactose; 3-FL, 3-fucosyllactose; LNT, Lacto-N-tetraose; LNnT, Lacto-N-neotetraose; 3′-SL, 3′-sialyllactose; 6′-SL, 6′-sialyllactose. Different superscript letters (a–e) indicated significant differences (adjusted *p* < 0.05 by the Bonferroni correction for multiple tests) according to an independent nonparametric test (Kruskal–Wallis one-way ANOVA, all pairwise).

Our data showed that in general, lactational stage and geographical location influenced the content of HMOs. The variation in all six HMOs over 0–13 months postpartum is shown in Figure 1 and Supplementary Table S1. The six HMOs varied in notable ways. For example, the dominant fucosylated HMO changed from 2′-FL to 3-FL, and the predominant sialylated HMOs changed from 6′-SL to 3′-SL due to differences in concentration over time. The prominent variation was observed in the content of 6′-SL, which demonstrates a pattern of initial increase followed by a subsequent decrease. Among the five lactational stages, the transitional milk has the highest concentration, which was 31 times greater than the concentration in mature milk at 300–400 days postpartum, where the content is the lowest.

**Figure 2:**
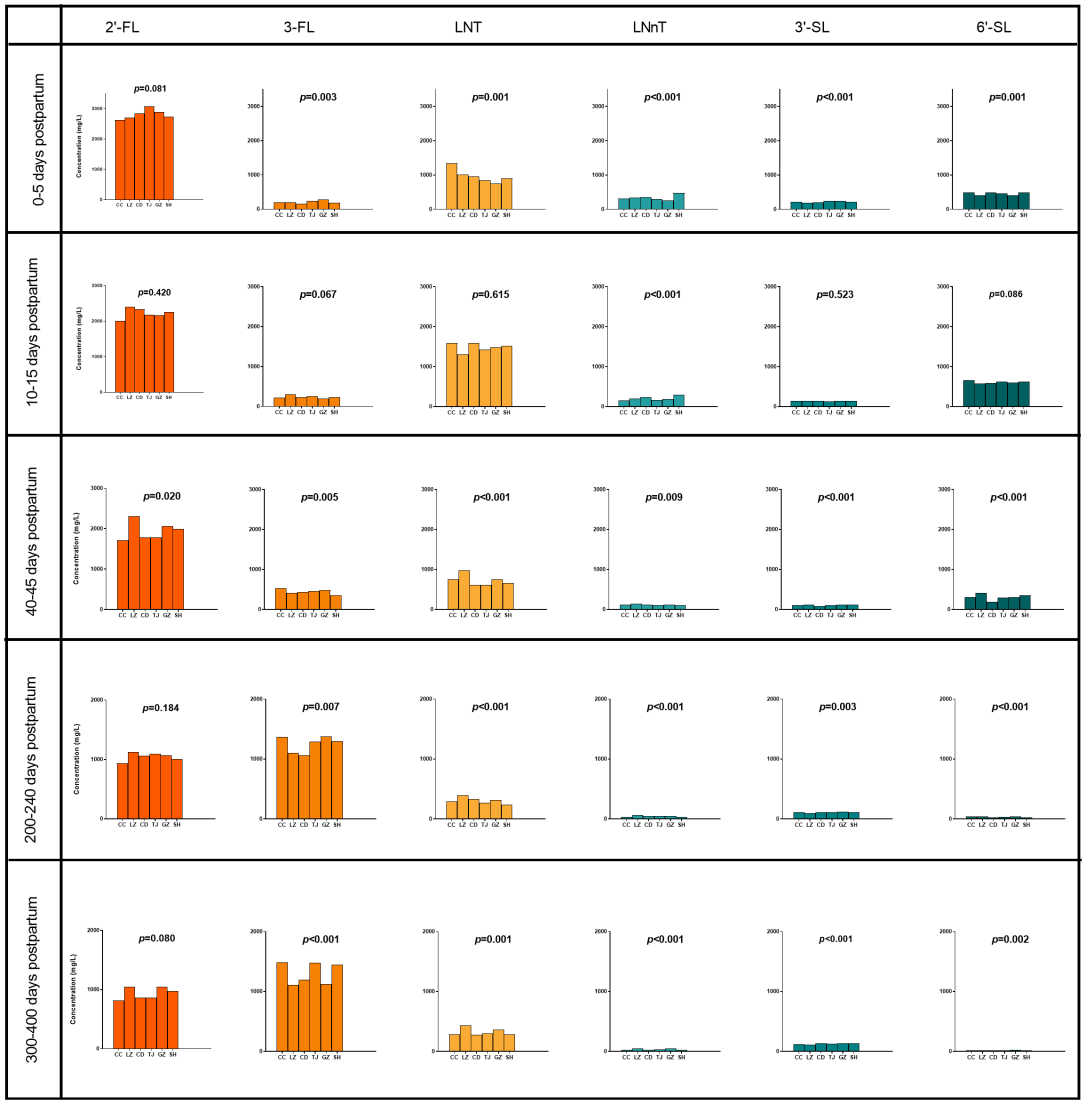
Concentration of six human milk oligosaccharides (HMOs) at six geographical sites at 0–13 months postpartum. 2′-FL, 2′-fucosyllactose; 3-FL, 3-fucosyllactose; LNT, Lacto-N-tetraose; LNnT, Lacto-N-neotetraose; 3′-SL, 3′-sialyllactose; 6′-SL, 6′-sialyllactose. Total HMO concentration was calculated as the sum of 2′-FL, 3-FL, LNT, LNnT, 3′-SL, and 6′-SL. CC, Changchun; LZ, Lanzhou; CD, Chengdu; TJ, Tianjin; GZ, Guangzhou; SH, Shanghai. Nonparametric test-Kruskal Wallis one-way ANOVA (multiple comparisons: all pairwise, adj-*p*).

Geographical location was associated with differences in the concentrations of HMOs (Figure 2; Supplementary Table S2). The content of 2′-FL in milk collected at 40–45 days postpartum in Lanzhou was about 1.3 times higher than in samples collected in Chengdu. At 40–45 days postpartum, the concentration of 3-FL in breast milk from Changchun was significantly higher compared to Shanghai, with levels of 526 mg/L and 350 mg/L, respectively. The breast milk from Lanzhou showed the significantly highest concentration of LNT and LNnT among the six sites at 40–240 days postpartum. Breast milk from coastal cities (Guangzhou and Shanghai) contained significantly higher concentrations of 3′-SL compared to inland cities (Lanzhou) at 200–400 days postpartum. The concentration of 6′-SL in breast milk from Chengdu is significantly higher compared to that in breast milk from Guangzhou in colostrum, with an increase of 21%.

### 3.3. The proportion of high and low 2’-FL level subjects

**Figure 3:**
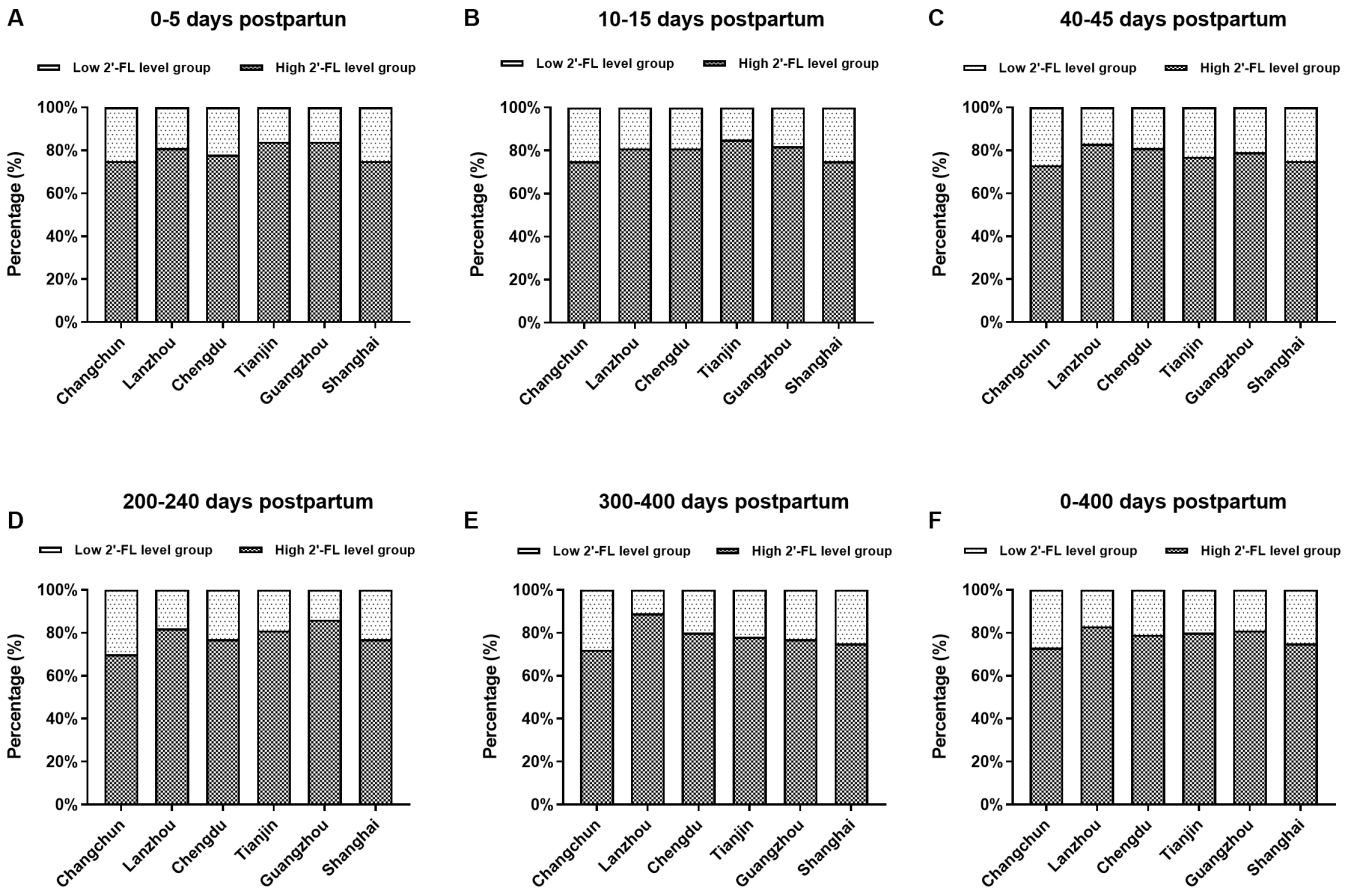
The proportion of high and low 2′-FL level groups among the six regions at 0–5 days postpartum **(A)**, 10–15 days postpartum **(B)**, 40–45 days postpartum **(C)**, 200–240 days postpartum **(D)**, 300–400 days postpartum **(E)**, and 0–400 days postpartum **(F)**.

2′-FL was detected in 99.8% (2,611/2,616) of the samples and a clear separation was found in the distribution of 2′-FL levels (Supplementary Figure S2). Milk samples with 2′-FL concentration greater than or equal to 200 mg/L were assigned to the high 2′-FL level group while those with 2′-FL concentration below 200 mg/L were assigned to the low 2′-FL level group. The distribution of high and low 2′-FL subjects in China was determined by testing samples from six geographical regions. The proportion of high and low 2′-FL level subjects at the 6 geographical sites and at each sampling time is shown in Figures 3A–E. The fraction of low 2′-FL level subjects was lower than the proportion of high 2′-FL level subjects at every geographical site. There was no significant difference in the proportion of high and low 2′-FL level subjects among the six regions at all five sampling time points (*p* = 0.697, 0–5 days postpartum; *p* = 0.655, 10–15 days postpartum; *p* = 0.598, 40–45 days postpartum; *p* = 0.116, 200–240 days postpartum; *p* = 0.136, 300–400 days postpartum). The percentage of high and low 2′-FL level subjects in China is 79%: 21% (Figure 3F).

### 3.4. Variations in HMO concentration over lactational stage and geographical sites stratified by high and low 2’-FL level

The median (p25, p75) values of six HMOs in the high and low 2′-FL level groups were 2′-FL 1,849 (1,166, 2,656) mg/L and 22 (11, 40) mg/L, 3-FL 458 (217, 1,078) mg/L and 1,401 (826, 2,033) mg/L, LNT 514 (285, 921) mg/L and 882 (403, 1,759) mg/L, LNnT 114 (52, 222) mg/L and 42 (16, 108) mg/L,3′-SL 126 (101, 166) mg/L and 123 (97, 161) mg/L, 6′-SL 187 (29, 460) mg/L and 195 (29, 463) mg/L. The median (p25, p75) values of total six HMOs in the high and low 2′-FL level groups were 3,639 (2,947, 4,917) mg/L and 3,062 (2,566, 3,695) mg/L, respectively.

**Figure 4:**
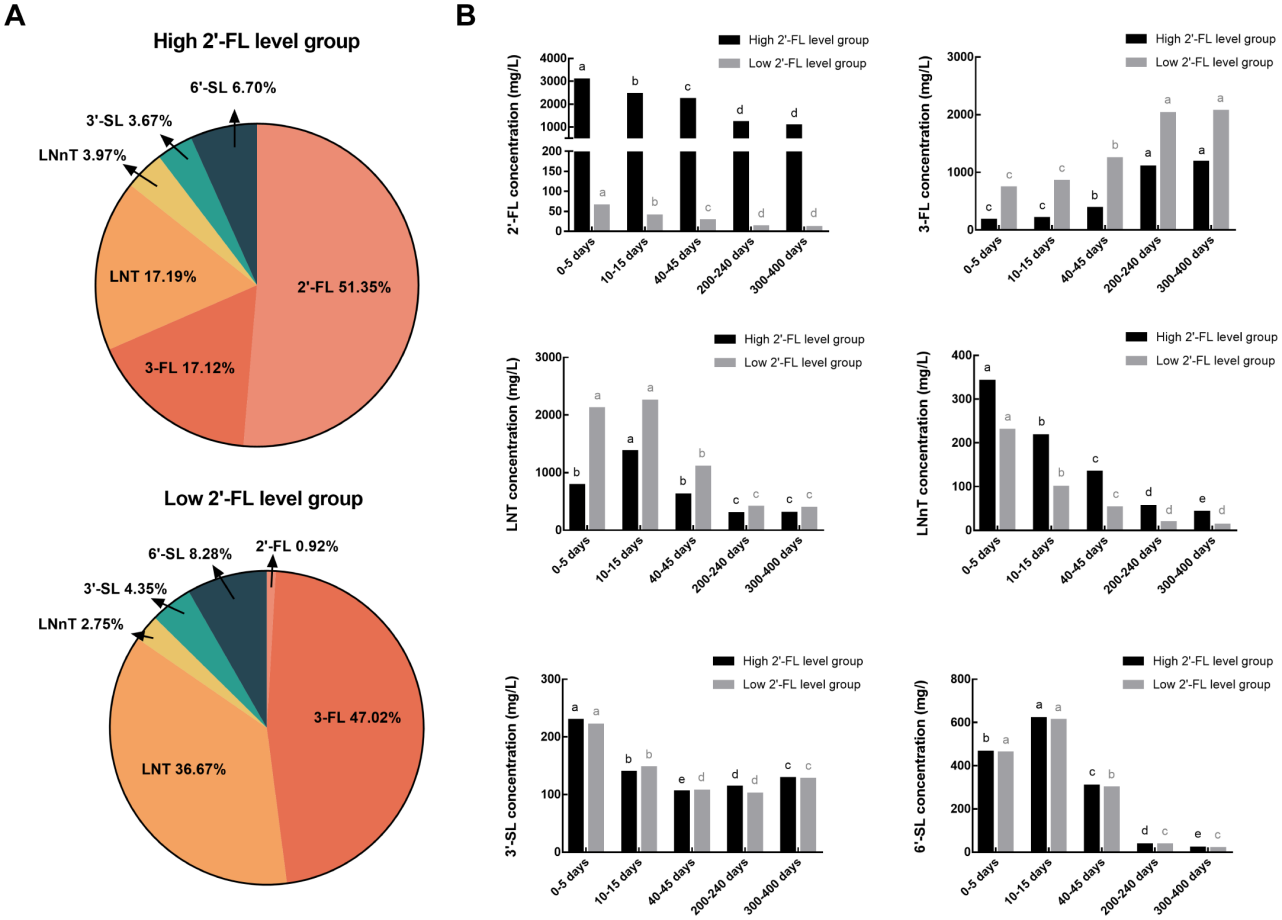
The proportion of single HMOs **(A)** and the variations of six HMOs from 0–13 months postpartum **(B)** in the high and low 2′-FL level groups. 2′-FL, 2′-fucosyllactose; 3-FL, 3-fucosyllactose; LNT, Lacto-N-tetraose; LNnT, Lacto-N-neotetraose; 3′-SL, 3′-sialyllactose; 6′-SL, 6′-sialyllactose. Total HMO concentration was calculated as the sum of 2′-FL, 3-FL, LNT, LNnT, 3′-SL, and 6′-SL.

The proportion of individual HMOs and the variation in each of the six HMOs from 0–13 months postpartum are separated by high and low 2′-FL level categorization and shown in Figure 4. Although the concentration of fucosylated and acetylated HMOs differed significantly between the high and low 2′-FL level groups, the lactational variation of each HMO in both groups were consistent with those before grouping. And the effect of geographical variation on HMO concentration was also consistent with before grouping, except for LNT and LNnT, which were not influenced by geographic factors in the low 2′-FL level group (Tables 2, 3).

**Table 2:** The concentration of human milk oligosaccharides (HMOs) at six geographical sites over 0–400 days postpartum in high 2’-FL level groups [Median (p25, p75)] (mg/L).

HMOs	Lactational stages	Geographical sites						*p* value
		Changchun	Lanzhou	Chengdu	Tianjin	Guangzhou	Shanghai	
2′-FL	0–5 days	2,895 (2,471, 3,447) ^c^	2,831 (2,162, 3,528) ^c^	3,296 (2,552, 3,900) ^a,b^	3,571 (3,017, 4,572) ^a^	3,263 (2,551, 5,222) ^a,b^	2,838 (2,301, 3,765) ^b^	<0.001
	10–15 days	2,271 (1,833, 2,776)	2,564 (2,167, 2,985)	2,472 (2 065, 2,875)	2,504 (2,080, 2,900)	2,446 (1,934, 2,922)	2,432 (1,965, 3,222)	0.661
	40–45 days	2,201 (1,678, 2,683) ^a,b^	2,490 (2,162, 2,926) ^a^	1,958 (1,579, 2,326) ^b^	2,148 (1,644, 2,614) ^b^	2,313 (1,803, 2,941) ^a,b^	2,159 (1,627, 2,764) ^a,b^	<0.001
	200–240 days	1,153 (905, 1,633)	1,421 (923, 1,833)	1,272 (896, 1,742)	1,228 (875, 1,556)	1,238 (935, 1,671)	1,071 (802, 1,424)	0.081
	300–400 days	1,019 (775, 1,507) ^a,b^	1,243 (916, 1,673) ^a^	945 (695, 1,486) ^b^	1,030 (686, 1,364) ^a,b^	1,183 (896, 1,607) ^a,b^	1,073 (843, 1,385) ^a,b^	0.008
3-FL	0–5 days	150 (98, 220) ^a,b^	169 (115, 263) ^a,b^	133 (100, 209) ^b^	176 (117, 294) ^a,b^	198 (132, 326) ^a^	148 (93, 225) ^b^	0.004
	10–15 days	192 (125, 281) ^b^	266 (188, 411) ^a^	209 (146, 284) ^a,b^	206 (133, 295) ^b^	170 (121, 254) ^b^	209 (93, 290) ^b^	0.002
	40–45 days	433 (320, 581) ^a^	338 (200, 482) ^b^	388 (311, 560) ^a,b^	375 (230, 501) ^a,b^	397 (289, 597) ^a,b^	298 (193, 505) ^b^	<0.001
	200–240 days	1,047 (757, 1,516) ^a,b^	1,051 (707, 1,312) ^a,b^	991 (607, 1,277) ^b^	1,180 (891, 1,469) ^a,b^	1,148 (811, 1,553) ^a,b^	1,247 (908, 1,477) ^a^	0.007
	300–400 days	1,355 (964, 1,611) ^a^	1,018 (592, 1,374) ^b^	1,132 (808, 1,552) ^a,b^	1,373 (976, 1,616) ^a^	1,011 (712, 1,295) ^b^	1,233 (878, 1,611) ^a,b^	<0.001
LNT	0–5 days	1,150 (757, 1,487) ^a^	829 (464, 1,304) ^a,b^	918 (667, 1,218) ^a,b^	627 (438, 1,089) ^b^	538 (256, 1,061) ^b^	810 (425, 1,355) ^a,b^	<0.001
	10–15 days	1,495 (1,166, 2,018)	1,230 (818, 1,691)	1,463 (990, 1,670)	1,299 (967, 1,630)	1,393 (962, 1,770)	1,365 (1,074, 1,865)	0.450
	40–45 days	682 (471, 943) ^a,b^	851 (580, 1,287) ^a^	581 (429, 798) ^b^	530 (346, 781) ^b^	617 (449, 904) ^b^	583 (392, 782) ^b^	<0.001
	200–240 days	260 (182, 423) ^b^	389 (272, 530) ^a^	330 (214, 500) ^a,b^	255 (199, 379) ^b^	287 (193, 395) ^b^	238 (172, 350) ^b^	<0.001
	300–400 days	245 (151, 418) ^b^	419 (228, 537) ^a^	272 (174, 401) ^b^	297 (179, 466) ^a,b^	329 (226, 456) ^a,b^	277 (171, 425) ^b^	0.002
LNnT	0–5 days	319 (262, 421) ^b^	350 (271, 427) ^b^	400 (294, 533) ^a,b^	295 (244, 416) ^b^	272 (194, 407) ^b^	499 (364, 615) ^a^	<0.001
	10–15 days	186 (133, 238) ^b^	216 (146, 276) ^b^	250 (171, 364) ^a,b^	191 (137, 282) ^b^	209 (140, 269) ^b^	305 (242, 392) ^a^	<0.001
	40–45 days	133 (96, 194) ^a,b^	155 (115, 212) ^a^	130 (86, 175) ^b^	116 (73, 165) ^b^	142 (85, 198) ^a,b^	119 (77, 165) ^b^	0.002
	200–240 days	50 (28, 74) ^b^	85 (48, 126) ^a^	52 (28, 93) ^b^	59 (37, 102) ^a,b^	53 (26, 103) ^b^	39 (25, 68) ^b^	<0.001
	300–400 days	36 (13, 66) ^b^	62 (34, 99) ^a^	32 (13, 72) ^b^	41 (20, 82) ^a,b^	57 (25, 90) ^a,b^	30 (17, 60) ^b^	<0.001
3′-SL	0–5 days	216 (175, 252) ^a,b^	195 (137, 260) ^b^	204 (167, 244) ^b^	249 (201, 316) ^a^	246 (207, 324) ^a^	223 (168, 282) ^a,b^	<0.001
	10–15 days	144 (133, 166)	132 (101, 158)	138 (124, 164)	132 (115, 160)	140 (123, 162)	135 (119, 154)	0.368
	40–45 days	104 (86, 124) ^a,b^	109 (97, 131) ^a,b^	83 (72, 99) ^c^	104 (84, 119) ^b^	112 (95, 132) ^a,b^	115 (100, 136) ^a^	<0.001
	200–240 days	117 (101, 142) ^a^	100 (83, 125) ^b^	109 (88, 135) ^a,b^	113 (91, 142) ^a,b^	119 (98, 139) ^a^	110 (98, 132) ^a,b^	0.013
	300–400 days	124 (101, 150)	116 (97, 150)	134 (108, 177)	135 (100, 165)	131 (110, 160)	133 (110, 170)	0.099
6′-SL	0–5 days	490 (416, 584) ^a^	408 (349, 543) ^a,b^	475 (420, 548) ^a,b^	465 (389, 556) ^a,b^	404 (299, 501) ^b^	490 (420, 560) ^a,b^	0.004
	10–15 days	660 (555, 795) ^a^	568 (454, 699) ^a,b^	584 (442, 657) ^b^	640 (497, 763) ^a,b^	611 (519, 783) ^a,b^	630 (540, 758) ^a,b^	0.034
	40–45 days	290 (210, 406) ^b^	402 (320, 491) ^a^	183 (139, 269) ^c^	294 (181, 406) ^b^	300 (216, 374) ^b^	363 (277, 484) ^a^	<0.001
	200–240 days	41 (20, 59) ^a,b^	44 (28, 69) ^a^	28 (20, 47) ^b^	33 (21, 52) ^a,b^	36 (22, 54) ^a,b^	25 (16, 53) ^b^	0.001
	300–400 days	22 (11, 34) ^a,b^	18 (11, 28) ^b^	15 (10, 23) ^b^	21 (13, 38) ^a,b^	26 (15, 42) ^a^	20 (13, 40) ^a,b^	0.001
Total six HMOs	0–5 days	5,445 (5,032, 5,923) ^a,b^	4,936 (4,681, 5,566) ^b^	5,497 (4,978, 6,096) ^a,b^	5,802 (5,190, 6,533) ^a^	5,439 (4,871, 6,794) ^a^	5,389 (4,905, 5,919) ^a,b^	<0.001
	10–15 days	5,063 (4,705, 5,381)	5,182 (4,742, 5,729)	5,205 (4,891, 5,460)	5,173 (4,498, 5,612)	5,128 (4,691, 5,583)	5,314 (4,906, 5,950)	0.285
	40–45 days	3,909 (3,600, 4,308) ^c^	4,492 (4,146, 5,025) ^b^	3,528 (3,166, 3,736) ^d^	3,756 (3,119, 4,191) ^c,d^	4,179 (3,704, 4,545) ^a^	3,854 (3,338, 4,491) ^c^	<0.001
	200–240 days	2,918 (2,672, 3,342) ^a,b^	3,156 (2,820, 3,422) ^a^	2,915 (2,617, 3,202) ^b^	2,931 (2,752, 3,176) ^a,b^	3,095 (2,748, 3,358) ^a,b^	2,803 (2,622, 3,075) ^b^	<0.001
	300–400 days	2,966 (2,718, 3,312)	3,016 (2,739, 3,294)	2,813 (2,506, 3,133)	2,934 (2,639, 3,245)	2,908 (2,689, 3,141)	2,919 (2,745, 3,200)	0.081

^a–d^Values within a row in individual cities with different superscript letters were significantly different (adjusted *p* < 0.05 by the Bonferroni correction for multiple tests) according to an independent nonparametric test (Kruskal–Wallis one-way ANOVA, all pairwise). 2′-FL, 2′-fucosyllactose; 3-FL, 3-fucosyllactose; LNT, Lacto-N-tetraose; LNnT, Lacto-N-neotetraose; 3′-SL, 3′-sialyllactose; 6′-SL, 6′-sialyllactose. Total HMO concentration was calculated as the sum of 2′-FL, 3-FL, LNT, LNnT, 3′-SL, and 6′-SL.

**Table 3:** Concentration of human milk oligosaccharides (HMOs) at six geographical sites over 0–400 days postpartum in low 2’-FL level groups [Median (p25, p75)] (mg/L).

HMOs	Lactational stages	Geographical sites						*p* value
		Changchun	Lanzhou	Chengdu	Tianjin	Guangzhou	Shanghai	
2′-FL	0–5 days	64 (45, 79)	51 (46, 75)	59 (45, 74)	69 (54, 94)	64 (47, 86)	38 (21, 90)	0.467
	10–15 days	37 (32, 43)	40 (30, 42)	40 (35, 46)	38 (28, 50)	39 (36, 47)	40 (30, 53)	0.962
	40–45 days	25 (21, 32) ^b^	37 (33, 43) ^a^	29 (22, 35) ^a,b^	30 (24, 41) ^a,b^	14 (5, 20) ^c^	23 (20, 29) ^b,c^	<0.001
	200–240 days	14 (9, 17) ^a,b^	14 (10, 18) ^a,b^	11 (9, 16) ^a,b^	16 (12, 22) ^a^	15 (2, 20) ^a,b^	8 (6, 12) ^b^	0.012
	300–400 days	8 (5, 10) ^b^	12 (8, 20) ^a,b^	14 (10, 26) ^a,b^	16 (11, 22) ^a^	12 (7, 13) ^a,b^	7 (4, 10) ^b^	0.001
3-FL	0–5 days	756 (480, 947)	518 (385, 818)	728 (394, 1,114)	714 (577, 962)	944 (674, 1,302)	721 (421, 1,138)	0.190
	10–15 days	933 (647, 1,187)	1,013 (819, 1,408)	944 (547, 1,605)	813 (649, 1,073)	758 (661, 998)	1,064 (561, 1,115)	0.630
	40–45 days	1,299 (826, 1,692) ^a,b^	1,305 (841, 1,618) ^a,b^	1,170 (774, 1,692) ^a,b^	1,090 (854, 1,460) ^a,b^	1,641 (1,192, 1,907) ^a^	1,075 (782, 1,462) ^b^	0.035
	200–240 days	1,945 (1,514, 2,217)	2,195 (1,468, 2,454)	1,991 (1,264, 2,165)	2,163 (1,387, 2,474)	2,336 (1,814, 2,743)	1,930 (1,627, 2,558)	0.181
	300–400 days	2,131 (1,590, 2,567)	2,041 (1,540, 2,628)	1,996 (1,483, 2,405)	2,050 (1,687, 2,361)	1,896 (1,270, 2,403)	2,367 (1,892, 2,757)	0.450
LNT	0–5 days	2,126 (1,670, 2,445)	2,115 (1,415, 2,644)	2,488 (1,495, 3,150)	2,052 (1,443, 2,557)	2,021 (1,067, 2,434)	2,065 (1,764, 2,332)	0.790
	10–15 days	2,030 (1,115, 2,331)	2,037 (1,947, 2,700)	2,298 (1,499, 2,787)	2,241 (1,808, 2,622)	2,299 (1,712, 2,759)	3,121 (2,124, 3,852)	0.072
	40–45 days	1,005 (837, 1,294)	1,446 (957, 1,769)	803 (583, 1,216)	977 (800, 1,366)	1,104 (805, 1,314)	1,184 (883, 1,962)	0.064
	200–240 days	318 (222, 536)	508 (354, 806)	417 (246, 543)	383 (176, 606)	443 (280, 648)	345 (154, 842)	0.177
	300–400 days	340 (271, 541)	489 (285, 785)	344 (242, 617)	336 (253, 599)	671 (353, 785)	363 (185, 495)	0.055
LNnT	0–5 days	234 (159, 458)	208 (134, 400)	219 (120, 373)	273 (205, 372)	211 (153, 269)	236 (164, 396)	0.556
	10–15 days	76 (56, 129)	75 (51, 142)	73 (41, 238)	101 (60, 152)	115 (70, 164)	88 (62, 261)	0.733
	40–45 days	41 (28, 81)	56 (36, 115)	44 (28, 67)	62 (39, 100)	49 (29, 88)	56 (34, 82)	0.407
	200–240 days	14 (6, 23)	23 (17, 41)	18 (11, 38)	20 (13, 35)	19 (12, 40)	10 (4, 18)	0.083
	300–400 days	7 (4, 15)	15 (6, 27)	7 (6, 22)	14 (7, 27)	20 (10, 34)	11 (5, 18)	0.051
3′-SL	0–5 days #	169 (147, 242)	167 (108, 250)	218 (172, 251)	240 (220, 301)	216 (197, 295)	215 (203, 222)	0.029
	10–15 days	142 (121, 167)	156 (130, 198)	159 (147, 172)	142 (116, 169)	141 (124, 175)	145 (134, 163)	0.462
	40–45 days	93 (78, 115) ^b^	118 (99, 146) ^a^	95 (72, 104) ^b^	99 (76, 125) ^a,b^	111 (91, 135) ^a,b^	113 (104, 128) ^a,b^	0.001
	200–240 days	98 (89, 122) ^a,b^	90 (74, 108) ^b^	99 (88, 145) ^a,b^	100 (84, 116) ^a,b^	103 (90, 126) ^a,b^	117 (101, 150) ^a^	0.043
	300–400 days	113 (93, 138) ^b^	104 (87, 124) ^b^	137 (107, 145) ^a,b^	126 (90, 143) ^a,b^	144 (127, 170) ^a^	140 (114, 179) ^a,b^	0.001
6′-SL	0–5 days	493 (398, 552)	427 (285, 576)	588 (404, 721)	452 (397, 507)	431 (312, 561)	530 (376, 607)	0.454
	10–15 days	654 (558, 763)	630 (537, 765)	666 (544, 738)	602 (474, 742)	568 (512, 734)	566 (509, 772)	0.869
	40–45 days	305 (251, 428) ^a,b^	425 (296, 551) ^a^	228 (150, 274) ^b^	267 (163, 382) ^b^	299 (219, 365) ^a,b^	325 (212, 451) ^a,b^	0.001
	200–240 days #	40 (20, 73)	43 (33, 86)	29 (20, 37)	29 (10, 45)	44 (34, 64)	28 (14, 40)	0.015
	300–400 days	16 (9, 33)	29 (18, 58)	19 (13, 29)	17 (7, 27)	21 (15, 30)	19 (12, 34)	0.241
Total six HMOs	0–5 days	3,953 (3,211, 4,852)	3,534 (3,128, 4,334)	4,261 (3,676, 4,846)	3,882 (3,579, 4,191)	3,678 (3,416, 4,264)	3,960 (3,682, 4,052)	0.535
	10–15 days	3,723 (3,140, 4,255) ^b^	4,269 (3,693, 4,858) ^a,b^	4,125 (4,002, 4,846) ^a,b^	4,095 (3,570, 4,568) ^a,b^	3,912 (3,598, 4,428) ^a,b^	4,643 (4,108, 5,880) ^a^	0.039
	40–45 days	2,979 (2,466, 3,175) ^a,b^	3,444 (2,773, 3,832) ^a^	2,506 (2,143, 2,990) ^b^	2,639 (2,412, 3,084) ^b^	3,271 (2,968, 3,480) ^a^	2,846 (2,503, 3,295) ^a,b^	<0.001
	200–240 days	2,388 (2,114, 2,909) ^b^	2,936 (2,403, 3,234) ^a,b^	2,670 (2,057, 2,786) ^b^	2,807 (2,317, 3,097) ^a,b^	2,938 (2,733, 3,418) ^a^	2,738 (2,180, 3,062) ^a,b^	0.005
	300–400 days	2,591 (2,193, 3,068)	2,860 (2,629, 3,190)	2,542 (2,152, 3,136)	2,687 (2,451, 3,072)	2,789 (2,236, 3,216)	2,884 (2,527, 3,179)	0.540

^a–c^Values within a row in individual cities with different superscript letters were significantly different (adjusted *p* < 0.05 by the Bonferroni correction for multiple tests) according to an independent nonparametric test (Kruskal–Wallis one-way ANOVA, all pairwise). 2′-FL, 2′-fucosyllactose; 3-FL, 3-fucosyllactose; LNT, Lacto-N-tetraose; LNnT, Lacto-N-neotetraose; 3′-SL, 3′-sialyllactose; 6′-SL, 6′-sialyllactose. Total HMO concentration was calculated as the sum of 2′-FL, 3-FL, LNT, LNnT, 3′-SL, and 6′-SL.

## 4. Discussion

In this study, we analyzed the concentration of six major HMOs in 2,616 human milk samples collected from six geographical sites in China, including the northeast, northwest, southwest, east, south, and a metropolitan city, spanning from the initiation of lactation up to 13 months postpartum. Despite the samples being collected from different locations, standardized sample processing techniques and HPAEC-PAD-based analytical methods were uniformly employed. This multicenter study displayed the dynamic change in the concentrations of individual HMOs in breast milk collected from multiple sites and covering the first 13 months of lactation. Our results provide a comparative representation of 6 HMO concentration in the breast milk of Chinese mothers. This data provides a scientific basis for the fortification of this 6 HMOs in infant formula and serves as a reference for comparing HMO content in China.

**Figure 5:**
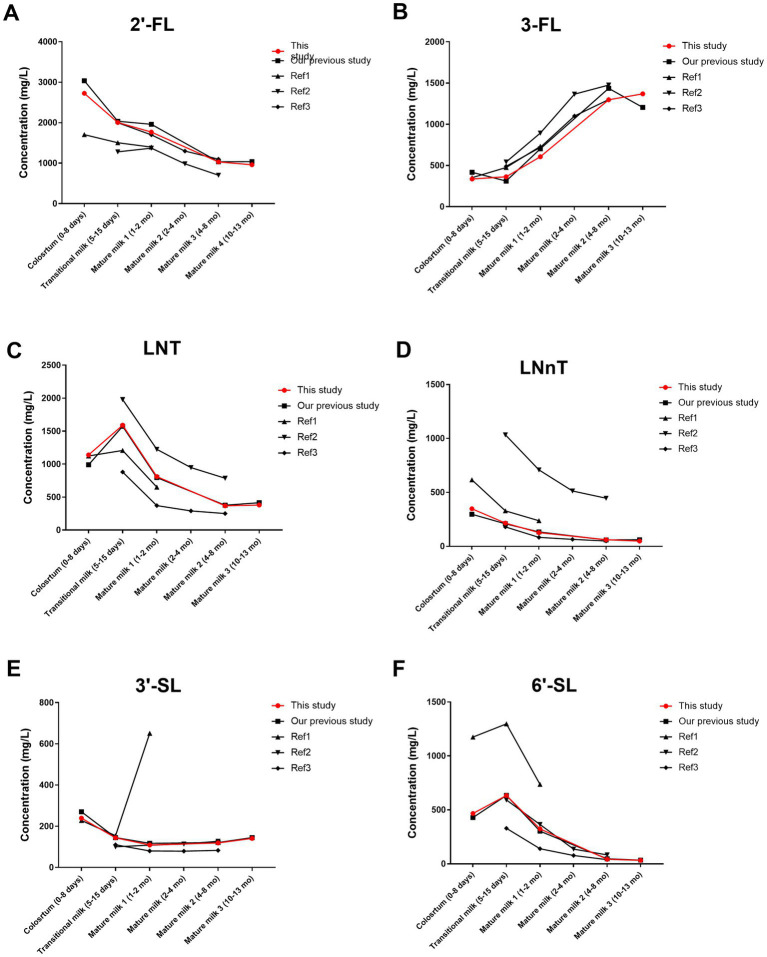
Comparison of HMO concentrations in this study and other studies in Chinese populations **(A–F)**. Our previous study (37); Ref 1, reference 1 (41); Ref 2, reference 2 (26); Ref 3, reference 3 (10). 2′-FL, 2′-fucosyllactose; 3-FL, 3-fucosyllactose; LNT, Lacto-N-tetraose; LNnT, Lacto-N-neotetraose; 3′-SL, 3′-sialyllactose; 6′-SL, 6′-sialyllactose. Total HMOs concentration was calculated as the sum of 2′-FL, 3-FL, LNT, LNnT, 3′-SL, and 6′-SL. The data points represent the average value.

Our findings contribute additional data on HMO content in colostrum, supplement the available literature on HMO content in breast milk up to one year postpartum, and enhance the comprehensive characterization of HMO content in breast milk within China (Figure 5). Ren et al. (39) reported the concentration of 24 HMOs at three lactational stages (0–6 days, 7–14 days, and 15–340 days postpartum). When comparing the variation trend of individual HMO during lactation as observed by Ren et al., we noticed a difference in the variation trend of 3′-SL. Ren et al. reported a gradual decrease in the concentration of 3′-SL throughout the sampling period. However, our observations revealed a U-shaped pattern in the variation of 3′-SL content across five lactational stages. Specifically, the concentration of 3′-SL in breast milk at 300–400 days postpartum (127 mg/L) was significantly 15% higher than that in breast milk at 200–240 days postpartum (110 mg/L). The study conducted by Zhang et al. (40). reported the content of HMOs in 203 breast milk samples from mothers in 8 cities in China at 15–180 days postpartum. The average concentration of 2′-FL in breast milk at 6 months postpartum was 1,180 mg/L. In our study, we found that the average concentration of 2′-FL in breast milk was 1,032 mg/L at 6–8 months postpartum. And the average concentration of 2′-FL was 961 mg/L at 10–13 months postpartum. Compared with our previous study (37), the number of geographical locations where samples were collected was increased from one to six. The approach accounts for geographic variation and is therefore more representative of the concentrations of HMOs in the breast milk of Chinese mothers. In addition, the variation in 3-FL concentration across the period of lactation was clarified for the first time. This study and our previous study both report that 3-FL increased during lactation, although there was no significant difference between 200–240 days and 300–400 days postpartum. In previous studies, 3-FL was present at 1,421 mg/L and 1,128 mg/L in milk at 200–240 days and 300–400 days postpartum, respectively. However, in this study with an enlarged sample size, the concentrations of 3-FL were 1,230 mg/L and 1,325 mg/L at 200–240 days and 300–400 days postpartum, respectively. 2′-FL was more abundant and 3-FL was less abundant at 0–45 days postpartum compared to other Chinese studies (10, 26, 41). This may be due to the earlier sampling time in this study, as 2′-FL decreased while 3-FL increased during lactation. Other HMOs were within the range reported by multiple previous studies (10, 26, 42, 43).

In comparing the HMOs content of Chinese breast milk with studies conducted in other countries and regions, Chinese mothers have higher levels of 3-FL in their breast milk collected at 4 months after postpartum. However, the concentration of LNT and LNnT in Chinese breast milk appeared to be lower compared to that observed in other countries and regions (Supplementary Table S3) (26, 27, 44–46). At 4–8 months postpartum, the concentration of 3-FL in this study was 1,230 mg/L, compared to 1,074 mg/L in Europe (46), 1,194 mg/L in the UAE (27), and 1,146 mg/L in Malaysia (26). At 10–13 months postpartum, the concentration of 3-FL in this study was 1,325 mg/L, compared to 1,138 mg/L in Malaysia (26). Additionally, it is worth noting that 3-FL was detected in 99.9% of the samples in this study which means 3-FL was ubiquitously present in the breast milk of Chinese mothers. This indicates that 3-FL in Chinese breast milk might have an important function in the health outcomes of Chinese infants. 3-FL is one of the few HMOs known to increase significantly during the course of lactation (12, 47, 48). This suggests an important physiological role of 3-FL and warrants further investigation. The concentration of LNT in the breast milk was 701 mg/L at 40–45 days postpartum in this study, 876 mg/L at 30 ± 3 days postpartum in Europe (46) and 1,026 mg/L at 28–50 days postpartum in Brazil (44). The concentration of LNnT in this study was 215 mg/L at 10–15 days postpartum and 61 mg/L at 200–240 days postpartum, respectively. In another study conducted in the United Arab Emirates (27), LNnT was found to be 765 mg/L at 5–15 days postpartum and 250 mg/L at 180 days postpartum, respectively. Furthermore, LNnT was more abundant in milk from Brazilian mothers at 28–50 days postpartum (198 mg/L) (44) compared to the Chinese mothers at 40–45 days postpartum (113 mg/L) measured in this study.

The results of this study showed that 79% of Chinese mothers have the secretor phenotype and 21% express a non-secretor phenotype, although high and low 2′-FL level categories were used instead of the terms secretor and non-secretor in this study. Our study is not the first to use a cut off of 200 mg/L 2′-FL to determine secretor type. A study conducted by Wu et al. (43) in China (Guangzhou) reported that a 2′-FL concentration cut off of 200 mg/L separated secretors and non-secretors with 100% accuracy compared to serological tests. Wu et al. (43) reported that 2′-FL concentration in the breast milk of secretor’s mothers was 3,020 mg/L, 2,540 mg/L, 1,960 mg/L, and 1,280 mg/L at 3, 7, 42, and 168 days postpartum, respectively. In our study, 2′-FL concentration in secretor’s breast milks was 3,442 mg/L, 2,516 mg/L, 2,261 mg/L, 1,306 mg/L, and 1,228 mg/L at 0–5, 10–15, 40–45, 200–240, and 300–400 days postpartum, respectively. Our result from six geographical sites is similar to that of other studies conducted in China which reported 77% secretor phenotype in Guangzhou (43), 80% secretor phenotype in Hohhot (28) and 81% secretor phenotype in (Beijing, Xuchang and Suzhou) (38). However, the prevalence of secretor phenotypes among Chinese mothers differs from the prevalence worldwide (7, 12, 27–30, 44, 45, 49–51). For example, secretor phenotype prevalence is 87% in Finland (45), 89% in Brazil (44), 72% in Kenya (49), 74% in UAE (27), 66% in Spain (7) and 64% in Gambia (30). A recent study also reported that The proportion of mothers who are secretors in South Africa is higher compared to other cohorts (32). Our findings provide data on the prevalence of the secretor phenotype among Chinese mothers, and add to the total literature describing the frequency of the secretor phenotype worldwide.

HMO composition may have a significant effect on the function of breast milk. Studies over the past decade have revealed that the function of HMOs is to some extent related to their structure (18, 20, 22, 52, 53), and secretor and non-secretor milk may function differently in some ways. An observational study showed that infants who received secretor milk had a higher relative abundance of *Lactobacillus* in the gut at 6 months of age compared to non-secretor, while infants who received non-secretor’s milk exhibited higher relative abundances of *Bacteroides* than secretor (54). An *in vitro* study showed that secretor milk inhibits the growth of *Streptococcus agalactiae* by reducing the production of the *Streptococcus agalactiae* envelope, while non-secretor milk inhibits the growth of *Streptococcus agalactiae* by changing the morphology of the biofilm (55). Two observational studies indicated that specific milk oligosaccharide structures may associate with the fecal microbiome alteration of either term or preterm infants (56, 57). For term infants, those fed by secretor mother’s milk were reported to have quicker establishment of bifidobacterial-laden microbiota than non-secretor mother’s milk. For premature infants, those fed by non-secretor mother’s milk had higher levels of *Proteobacteria* and lower levels of *Firmicutes* than non-secretor mothers. In this study, the concentration of six HMOs was stratified according to high and low 2′-FL level categories within a cohort of Chinese mothers. The individual HMO concentrations measured in this study were comparable to a prior study conducted in China which also reported on HMO concentrations in milk at 3–168 days postpartum in secretor and non-secretor mothers (43). In addition, the proportions of the six HMOs in the high 2′-FL level group were as follows: 51% 2′-FL, 17% 3-FL, 17% LNT, 4% LNnT, 4% 3′-SL and 7% 6′-SL. The proportions of the six HMOs in the low 2′-FL level group were as follows: 1% 2′-FL, 47% 3-FL, 37% LNT, 2% LNnT, 4% 3′-SL and 8% 6′-SL. Thus, milk from the high and low 2′-FL level groups displayed distinct profiles of HMOs.

By using the same sample collection and HMO measurement methods, our data from six geographical sites demonstrated that geographical location has an effect on the concentration of HMOs in the Chinese milks. The effect of geographical location on HMO concentration in China has been previously investigated by Ren et al. (39). Their study reported no significant difference in the total content of 24 HMOs in breast milk from mothers in coastal and inland areas of China. However, in our current study, we analyzed the variations in individual HMO content among six regions at different lactation stages. We observed that breast milk from coastal cities (Guangzhou and Shanghai) exhibited significantly higher concentrations of 3′-SL compared to inland cities (Lanzhou) at 200–400 days postpartum. Global research has documented geographical factors affecting HMO concentration (13, 32). A study conducted by McGuire et al. (13) in 11 global cohorts with 410 breast milk samples confirmed that HMO concentrations and profiles vary geographically. 2′-FL concentration was 3.9 times higher in milk collected in the US (2,740 mg/L) than in milk collected in Ghana (700 mg/L) at 2 weeks-5 months postpartum. Ma et al. (26) analyzed a total of 243 breast milk samples from China (Guangzhou) and Malaysia and reported that the 2′-FL mean (SD) concentration in breast milk of Chinese (Guangzhou) and Malaysian mothers is 704.1 (752.4) mg/L and 1,002.8 (803.4) mg/L, respectively, at 180 days postpartum. 3-FL mean (SD) concentration in the breast milk of Chinese (Guangzhou) and Malaysian mothers is 1,475.8 (789.9) mg/L and 1,146.1 (868.5) mg/L, respectively at 180 days postpartum. In our study, the concentration ranges of 2′-FL and 3-FL were 935–1,123 mg/L and 1,061–1,375 mg/L, respectively, at 200–240 days postpartum. Compared with other studies, variation among different geographical sites within China is smaller than the variation observed between different countries.

We also observed that geographical variations in concentration were not consistent across the 5 sampling times. For example, the breast milk of mothers in Chengdu had higher levels of 6′-SL than Guangzhou at 0–5 days postpartum, however, the breast milk of mothers in Chengdu had lower levels of 6′-SL than Guangzhou at 300–400 days postpartum. The influence of geographic factors is complex as geographic location may include multiple aspects, such as ethnicity, maternal nutrition, and climate. Although China is a large country, the majority of the population in the six geographical sites involved in this study are of Han ethnicity (>90% of recruited subjects). In this case, differences occurred despite similar genetic backgrounds, suggesting that environmental factors may be important. A study conducted by Seferovic et al. (58) observed that compared to a high carbohydrate diet, a high-fat diet decreased the concentrations of sialylated HMOs. Seppo et al. (59) demonstrated that the concentrations of 3-FL and 3′-SL were higher in colostrum from mothers in the probiotic supplementation group, whereas difucosyllacto-N-hexaose, LNT, lacto-N-fucopentaose I, and 6′-SL were lower in mothers who received probiotic supplementation relative to a control group. A published study from our team showed that dietary vitamins, vegetables and metal elements are positively associated with HMO concentration using a mixed effect model with lactational stage and secretor status as the random effect (60). In addition, investigations by Davis et al. (50) revealed that mothers nursing in the dry season produced significantly more total HMOs compared to mothers nursing in the wet season. Therefore, maternal nutrition, and climate need to be further examined in order to explain the effect of geographical location on HMO concentration.

HMOs are a research hotspot of infant nutrition (61–66). Several studies have investigated the safety and tolerability of infant formula with added HMOs, including containing single (61, 64), dual (67, 68), or five types of HMOs (69, 70). Research to date, showed 3-FL increased through lactation, especially in Chinese populations, in which 3-FL is reported to be higher than in other counties. LNT, the most abundant acetylated HMO is globally reported to be at a higher level than LNnT in mature milk (26, 27, 44, 45, 71). The six types of HMOs analyzed in this study are among the first to potentially be added to infant formula in China. The data from Chinese breast milk presented in this study can serve as a reference for adding these HMOs into formula milk in China.

This study has some limitations. First, we were unable to report on the maternal Lewis status of Chinese mothers due to the lack of data on *α*1-4 linked HMOs. Several studies have classified mothers as Lewis-positive and Lewis-negative according to the amount of Lacto-N-fucopentaose II (LNFP II) in breast milk because LNFP II contains the *α*1-4 linkage (38, 72, 73). Second, we did not investigate the effect of maternal nutrition on HMO concentration in lactating mothers. Future studies are needed to reveal the relationship between HMO concentration and maternal nutrition.

In conclusion, HMOs are an abundant class of carbohydrates found in human milk and have been linked to developmental outcomes in infants, specifically cognition, the developing immune system, and the gut microbiome. We systematically analyzed the dynamic changes in six abundant HMOs in 2,616 breast milk samples from 1,758 healthy mothers at 0–13 months postpartum collected across six geographical sites in China. The concentrations and proportions of these six HMOs varied dynamically during lactation, but this variation was consistent across multiple geographical sites. We determined that 79% of breast milk was classified as containing high 2′-FL levels and 21% of breastmilk contained low 2′-FL levels in this large Chinese cohort. This analysis of the HMO composition of human milk samples collected from six sites in China provided the most extensive data set reported to date, which is helpful for comparison with HMO data worldwide and will provide a strong foundation of scientific principles to further guide infant formula fortification strategies. Future studies may determine the relationship between changes in HMO composition and maternal and infant health.

## Data availability statement

The original contributions presented in the study are included in the article/Supplementary material, further inquiries can be directed to the corresponding author.

## Ethics statement

The protocol was approved by the Ethics Committee of Tianjin hospital of ITCWM Nankai hospital. This study was registered in the China Clinical Trial Center (ChiCTR1800015387). The studies were conducted in accordance with the local legislation and institutional requirements. The participants provided their written informed consent to participate in this study.

## Author contributions

SL: Formal analysis, Software, Visualization, Writing – original draft. YM: Conceptualization, Investigation, Validation, Writing – original draft. JW: Data curation, Methodology, Validation, Writing – review & editing. FT: Methodology, Software, Visualization, Writing – review & editing. DH: Writing – original draft. XX: Investigation, Validation, Writing – review & editing. XL: Data curation, Supervision, Writing – review & editing. YZ: Resources, Supervision, Writing – review & editing. SW: Funding acquisition, Project administration, Writing – original draft.

## Funding

The authors declare financial support was received for the research, authorship, and/or publication of this article. This research was funded by Abbott Nutrition Research & Development Center, Shanghai, China.

## Conflict of interest

The authors declare that the research was conducted in the absence of any commercial or financial relationships that could be construed as a potential conflict of interest.

## Publisher’s note

All claims expressed in this article are solely those of the authors and do not necessarily represent those of their affiliated organizations, or those of the publisher, the editors and the reviewers. Any product that may be evaluated in this article, or claim that may be made by its manufacturer, is not guaranteed or endorsed by the publisher.
