# Supplementary material for: Lactational and geographical variation in the concentration of six oligosaccharides in Chinese breast milk: a multicenter study over 13 months postpartum
Source: Front Nutr. 2023 Sep 5;10:1267287. doi: 10.3389/fnut.2023.1267287 (PMC10508235; doi:10.3389/fnut.2023.1267287)
Supplement: Supplementary file 1 [file Data_Sheet_1.docx]

Supplementary Material

Lactational and Geographical Variation in the Concentration of Six Oligosaccharides in Chinese Breast Milk: A Multicenter Study over 13 Months Postpartum

Shuang Liu1†, Yingyi Mao2†, Jin Wang1, Fang Tian2, David R. Hill3, Xiaoying Xiong2, Xiang Li2, Yanrong Zhao2, Shuo Wang1*

*** Correspondence:** Shuo Wang: wangshuo@nankai.edu.cn

# Supplementary Figures and Tables

## Supplementary Figures


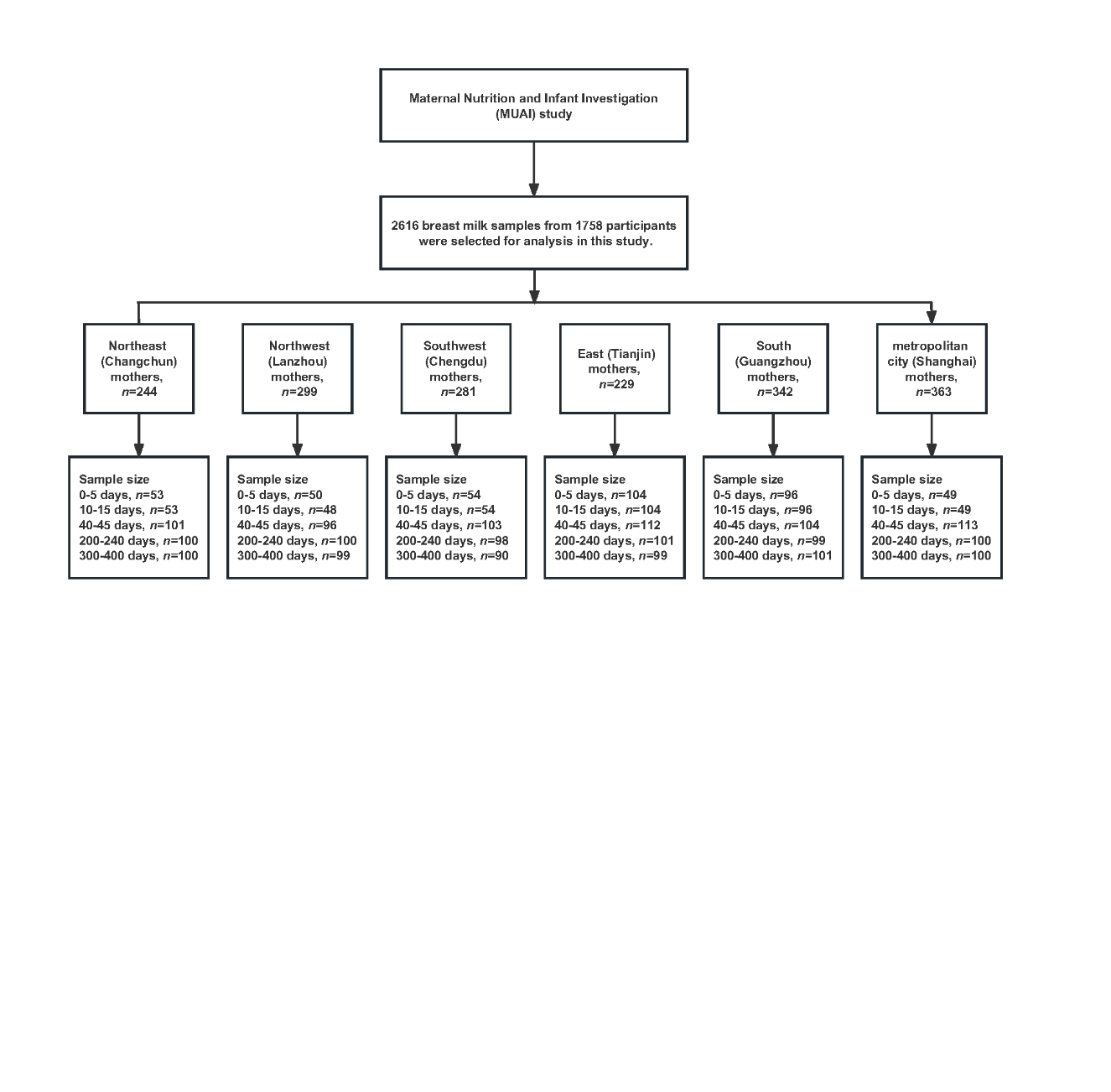


**Supplementary Figure 1.** Participant flow chart.


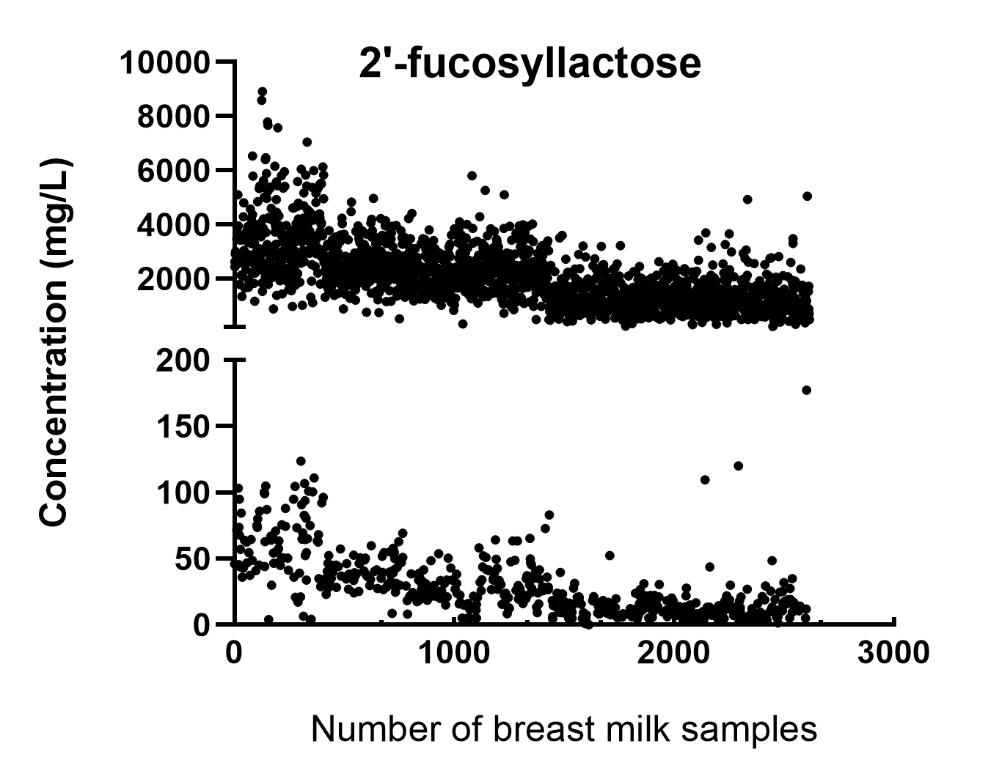


**Supplementary Figure 2.** The distribution of 2'-fucosyllactose concentration in 2616 breast milk samples.

## Supplementary Tables

**Supplementary Table 1.** Concentration of human milk oligosaccharides (HMOs) from 0 to 400 days postpartum (median (p25, p75)) (mg/L).

| HMOs | Lactation stages | | | | | *p* |
| --- | --- | --- | --- | --- | --- | --- |
|  | 0-5 days (n=406) | 10-15 days (n=404) | 40-45 days (n=629) | 200-240 days (n=599) | 300-400 days (n=578) |  |
| 2'-FL | 2865(1769, 3790) ^a^ | 2237(1422, 2805) ^b^ | 1916(1096, 2519) ^c^ | 1052(549, 1505) ^d^ | 935(457, 1369) ^d^ | <0.001 |
| 3-FL | 206(121, 401) ^c^ | 236(145, 437) ^c^ | 444(289, 755) ^b^ | 1230(872, 1649) ^a^ | 1325(892, 1777) ^a^ | <0.001 |
| LNT | 967(518, 1565) ^b^ | 1473(1070, 2034) ^a^ | 701(485, 1023) ^c^ | 300(205, 469) ^d^ | 317(205, 494) ^d^ | <0.001 |
| LNnT | 317(230, 444) ^a^ | 193(125, 281) ^b^ | 113(69, 166) ^c^ | 47(22, 84) ^d^ | 32(14, 66) ^e^ | <0.001 |
| 3'-SL | 228(182, 281) ^a^ | 140(120, 163) ^b^ | 106(87, 125) ^e^ | 110(92, 132) ^d^ | 127(104, 159) ^c^ | <0.001 |
| 6'-SL | 462(368, 556) ^b^ | 616(509, 754) ^a^ | 302(208, 421) ^c^ | 35(21, 55) ^d^ | 20(12, 34) ^e^ | <0.001 |
| Total 6 HMOs | 5183(4395, 5942) ^a^ | 5004(4446, 5498) ^a^ | 3705(3189, 4340) ^b^ | 2911(2642, 3234) ^c^ | 2903(2601, 3214) ^c^ | <0.001 |

^a,b,c,d,e^ Values within a row in individual HMOs with different superscript letters were significantly different (adjust *p*<0.05) according to an independent nonparametric test (Kruskal–Wallis one-way ANOVA, all pairwise). 2'-FL, 2'-fucosyllactose; 3-FL, 3-fucosyllactose; LNT, Lacto-N-tetraose; LNnT, Lacto-N-neotetraose; 3'-SL, 3'-sialyllactose; 6'-SL, 6'-sialyllactose. Total 6 HMOs concentration was calculated as the sum of 2'-FL, 3-FL, LNT, LNnT, 3'-SL, and 6'-SL.

**Supplementary Table 2.** Concentration of human milk oligosaccharides (HMOs) over geographical sites from 0 to 400 days postpartum (median (p25, p75)) (mg/L).

| HMOs | Geographical sites | Lactational stages | | | | |
| --- | --- | --- | --- | --- | --- | --- |
|  |  | 0-5 days | 10-15 days | 40-45 days | 200-240 days | 300-400 days |
| 2'-FL | Changchun | 2622(720,3244) | 1997(675,2604) | 1715(40,2489) ^a,b^ | 935(19,1474) | 815(16,1326) |
|  | Lanzhou | 2693(1908,3163) | 2393(1614,2923) | 2309(1355,2862) ^a^ | 1123(734,1727) | 1043(479,1455) |
|  | Chengdu | 2842(1863,3786) | 2330(1498,2828) | 1782(1253,2202) ^b^ | 1065(688,1572) | 865(616,1343) |
|  | Tianjin | 3072(344,4221) | 2172(176,2782) | 1781(174,2508) ^a,b^ | 1094(504,1464) | 866(49,1245) |
|  | Guangzhou | 2891(1586,4412) | 2160(1604,2824) | 2063(1346,2702) ^a,b^ | 1033(598,1589) | 1047(604,1505) |
|  | Shanghai | 2723(1932,3725) | 2246(1514,2905) | 1989(1061,2504) ^a,b^ | 1004(648,1379) | 977(518,1306) |
|  | *p* | 0.081 | 0.420 | 0.020 | 0.184 | 0.080 |
| 3-FL | Changchun | 198(113,436) ^a,b^ | 224(146,560) | 526(367,838) ^a^ | 1365(866,1846) ^a,b^ | 1487(1035,1813) ^a^ |
|  | Lanzhou | 195(125,275) ^a,b^ | 295(210,497) | 415(261,739) ^a,b^ | 1099(809,1542) ^a,b^ | 1133(764,1726) ^a,b^ |
|  | Chengdu | 151(101,299) ^b^ | 234(151,474) | 434(327,687) ^a,b^ | 1061(668,1448) ^b^ | 1191(817,1717) ^a,b^ |
|  | Tianjin | 242(124,468) ^a,b^ | 250(140,485) | 454(244,781) ^a,b^ | 1286(918,1688) ^a,b^ | 1478(1050,1951) ^a^ |
|  | Guangzhou | 272(153,562) ^a^ | 193(126,389) | 480(327,805) ^a,b^ | 1421(881,1962) ^a^ | 1107(760,1476) ^b^ |
|  | Shanghai | 180(95,315) ^b^ | 225(125,407) | 350(212,730) ^b^ | 1300(939,1604) ^a,b^ | 1449(1047,1891) ^a^ |
|  | *p* | 0.003 | 0.067 | 0.005 | 0.007 | <0.001 |
| LNT | Changchun | 1341(965,1916) ^a^ | 1587(1166,2066) | 747(525,1021) ^a,b^ | 291(202,434) ^b^ | 286(176,452) ^b^ |
|  | Lanzhou | 1006(572,1563) ^a,b^ | 1303(842,1943) | 978(613,1451) ^a^ | 397(274,593) ^a^ | 427(234,605) ^a^ |
|  | Chengdu | 951(714,1526) ^a,b^ | 1592(1005,1926) | 613(468,851) ^b^ | 332(224,528) ^a,b^ | 278(190,410) ^b^ |
|  | Tianjin | 843(490,1499) ^b^ | 1423(1091,2035) | 606(439,933) ^b^ | 265(198,397) ^b^ | 302(204,476) ^a,b^ |
|  | Guangzhou | 744(364,1445) ^b^ | 1478(1073,2042) | 748(486,1029) ^b^ | 314(203,475) ^a,b^ | 354(241,522) ^a,b^ |
|  | Shanghai | 903(489,1688) ^a,b^ | 1514(1150,2099) | 658(418,954) ^b^ | 240(172,371) ^b^ | 287(173,433) ^b^ |
|  | *p* | 0.001 | 0.615 | <0.001 | <0.001 | 0.001 |
| LNnT | Changchun | 313(230,422) ^b^ | 157(103,221) ^b^ | 111(62,172) ^a,b^ | 35(15,65) ^b^ | 22(6,47) ^b^ |
|  | Lanzhou | 345(249,426) ^b^ | 195(137,273) ^b^ | 141(98,187) ^a^ | 67(36,110) ^a^ | 47(19,80) ^a^ |
|  | Chengdu | 359(244,476) ^a,b^ | 238(144,353) ^a,b^ | 115(68,156) ^a,b^ | 45(21,85) ^b^ | 28(12,65) ^a,b^ |
|  | Tianjin | 291(233,400) ^b^ | 163(113,267) ^b^ | 103(61,156) ^b^ | 51(27,83) ^a,b^ | 34(14,64) ^a,b^ |
|  | Guangzhou | 255(185,404) ^b^ | 183(123,262) ^b^ | 117(68,182) ^a,b^ | 44(22,83) ^b^ | 50(19,77) ^a^ |
|  | Shanghai | 477(335,575) ^a^ | 293(213,381) ^a^ | 107(65,151) ^b^ | 35(18,63) ^b^ | 25(12,53) ^b^ |
|  | *p* | <0.001 | <0.001 | 0.009 | <0.001 | <0.001 |
| 3'-SL | Changchun | 212(166,249) ^b^ | 144(130,165) | 102(81,121) ^b^ | 114(96,132) ^a^ | 123(98,147) ^a,b^ |
|  | Lanzhou | 189(136,258) ^b^ | 140(102,165) | 111(98,133) ^a,b^ | 98(82,125) ^b^ | 112(93,143) ^b^ |
|  | Chengdu | 206(168,245) ^b^ | 144(126,166) | 84(72,100) ^c^ | 109(88,136) ^a,b^ | 135(109,175) ^a^ |
|  | Tianjin | 243(207,313) ^a^ | 133(115,163) | 103(81,119) ^b^ | 109(89,136) ^a,b^ | 131(96,159) ^a,b^ |
|  | Guangzhou | 241(199,319) ^a^ | 141(123,163) | 111(95,133) ^a,b^ | 117(96,136) ^a^ | 135(111,163) ^a^ |
|  | Shanghai | 221(170,270) ^a^ | 136(123,155) | 114(101,135) ^a^ | 113(99,135) ^a^ | 133(111,171) ^a^ |
|  | *p* | <0.001 | 0.523 | <0.001 | 0.003 | <0.001 |
| 6'-SL | Changchun | 493(417,572) ^a^ | 654(559,784) | 298(214,417) ^b^ | 41(20,67) ^a,b^ | 20(11,33) ^a,b^ |
|  | Lanzhou | 407(345,543) ^a,b^ | 576(464,704) | 408(308,516) ^a^ | 43(29,72) ^a^ | 19(12,31) ^a,b^ |
|  | Chengdu | 496(417,583) ^a^ | 592(460,665) | 187(140,270) ^c^ | 29(20,42) ^b^ | 15(10,25) ^b^ |
|  | Tianjin | 458(391,540) ^a,b^ | 627(475,759) | 288(181,404) ^b^ | 32(18,49) ^b^ | 19(10,35) ^a,b^ |
|  | Guangzhou | 409(306,528) ^b^ | 602(521,773) | 300(216,370) ^b^ | 39(23,56) ^a,b^ | 24(15,40) ^a^ |
|  | Shanghai | 494(394,559) ^a,b^ | 621(541,768) | 352(277,480) ^a,b^ | 25(16,51) ^b^ | 20(13,37) ^a,b^ |
|  | *p* | 0.001 | 0.086 | <0.001 | <0.001 | 0.002 |
| Total 6 HMOs | Changchun | 5398(4590,5784) | 4799(4371,5190) ^b^ | 3679(3230,4150) ^b^ | 2847(2464,3054) ^b^ | 2924(2580,3269) |
|  | Lanzhou | 4845(4330,5500) | 4961(4680,5579) ^a.b^ | 4317(3889,4976) ^a^ | 3083(2777,3353) ^a^ | 2973(2713,3257) |
|  | Chengdu | 5178(4561,6013) | 5156(4785,5440) ^a.b^ | 3413(2991,3700) ^c^ | 2858(2518,3148) ^b^ | 2809(2473,3126) |
|  | Tianjin | 5349(4337,6271) | 4820(4134,5541) ^b^ | 3482(2696,4060) ^b,c^ | 2911(2702,3166) ^a.b^ | 2894(2602,3229) |
|  | Guangzhou | 5120(4212,6387) | 4995(4385,5428) ^a.b^ | 3913(3468,4477) ^a,b^ | 3084(2743,3377) ^a^ | 2882(2639,3156) |
|  | Shanghai | 5183(4617,5642) | 5304(4716,5932) ^a^ | 3693(3170,4372) ^b^ | 2798(2611,3068) ^b^ | 2916(2647,3181) |
|  | *p* | 0.184 | 0.009 | <0.001 | <0.001 | 0.139 |

^a,b,c^ Values within a row in individual HMOs with different superscript letters were significantly different (adjust *p*<0.05) according to an independent nonparametric test (Kruskal–Wallis one-way ANOVA, all pairwise). 2'-FL, 2'-fucosyllactose; 3-FL, 3-fucosyllactose; LNT, Lacto-N-tetraose; LNnT, Lacto-N-neotetraose; 3'-SL, 3'-sialyllactose; 6'-SL, 6'-sialyllactose. Total 6 HMOs concentration was calculated as the sum of 2'-FL, 3-FL, LNT, LNnT, 3'-SL, and 6'-SL.

**Supplementary Table 3.** Comparison of six human milk oligosaccharides (HMOs) concentration in this study with studies in other countries and regions (MEDIAN (MEAN)) (mg/L).

| HMOs | Country | Mothers | Samples | Maternal phenotype | Lactational stages | | | | | | Reference |
| --- | --- | --- | --- | --- | --- | --- | --- | --- | --- | --- | --- |
|  |  |  |  |  | Colostrum (0-8 days) | Transitional milk (5-15 days) | Mature milk (1-2 months) | Mature milk (2-4 months) | Mature milk (4-8 months) | Mature milk (10-13 months) |  |
| 2'-FL | China (This study) | 1758 | 2618 | all | 2865 (2726) | 2237 (2007) | 1916 (1768) | - | 1052 (1032) | 935 (961) |  |
|  | Europe | 370 | 1491 | all | 3798 (3691) | 2630 (2627) | 2428 (2450) | 1728 (1819) | 1559 (1625) |  | (46) |
|  | UAE | 41 | 81 | all | - | 2021 | - | - | 997 | - | (27) |
|  | Brazil | 101 | 173 | all | 2462 | - | 2462 | 2081 | - | - | (44) |
|  | Finland | 802 | 802 | all | - | - | - | 2959 | - | - | (45) |
|  | Malaysia | 26 | 103 | all | 2249 | - | - | - | 1003 | 741 | (26) |
| 3-FL | China (This study) | 1758 | 2618 | all | 206 (336) | 236 (363) | 444 (606) | - | 1230 (1297) | 1325 (1369) |  |
|  | Europe | 370 | 1491 | all | 249 (422) | 368 (594) | 485 (720) | 937 (1140) | 1074 (1209) |  | (46) |
|  | UAE | 41 | 81 | all | - | 581 | - | - | 1194 | - | (27) |
|  | Brazil | 101 | 173 | all | 132 | - | 176 | 918 | - | - | (44) |
|  | Finland | 802 | 802 | all | - | - | - | 169 | - | - | (45) |
|  | Malaysia | 26 | 103 | all | 429 | - | - | - | 1146 | 1138 | (26) |
| LNT | China (This study) | 1758 | 2618 | all | 967 (1139) | 1473 (1590) | 701 (810) | - | 300 (368) | 317 (379) |  |
|  | Europe | 370 | 1491 | all | 685 (912) | 1080 (1213) | 876 (1009) | 493 (599) | 458 (526) |  | (46) |
|  | UAE | 41 | 81 | all | - | 1429 | - | - | 504 | - | (27) |
|  | Brazil | 101 | 173 | all | 991 | - | 1026 | 962 | - | - | (44) |
|  | Finland | 802 | 802 | all | - | - | - | 606 | - | - | (45) |
|  | Malaysia | 26 | 103 | all | 2393 | - | - | - | 867 | 1156 | (26) |
| LNnT | China (This study) | 1758 | 2618 | all | 317 (348) | 193 (215) | 113 (127) | - | 47 (61) | 32 (49) |  |
|  | Europe | 370 | 1491 | all | 294 (307) | 165 (177) | 142 (153) | 92 (108) | 83 (98) |  | (46) |
|  | UAE | 41 | 81 | all | - | 765 | - | - | 250 | - | (27) |
|  | Brazil | 101 | 173 | all | 410 | - | 198 | 234 | - | - | (44) |
|  | Finland | 802 | 802 | all | - | - | - | 696 | - | - | (45) |
|  | Malaysia | 26 | 103 | all | 1420 | - | - | - | 571 | 642 | (26) |
| 3'-SL | China (This study) | 1758 | 2618 | all | 228 (239) | 140 (144) | 106 (109) | - | 110 (119) | 127 (141) |  |
|  | Europe | 370 | 1491 | all | 240 (254) | 144 (149) | 135 (141) | 124 (130) | 126 (132) |  | (46) |
|  | UAE | 41 | 81 | all | - | 226 | - | - | 134 | - | (27) |
|  | Brazil | 101 | 173 | all | 203 |  | 241 | 342 | - | - | (44) |
|  | Finland | 802 | 802 | all | - | - | - | 321 | - | - | (45) |
|  | Malaysia | 26 | 103 | all | 222 | - | - | - | 135 | 158 | (26) |
| 6'-SL | China (This study) | 1758 | 2618 | all | 462 (467) | 616 (633) | 302 (324) | - | 35 (43) | 20 (33) |  |
|  | Europe | 370 | 1491 | all | 535 (543) | 636 (649) | 452 (465) | 133 (151) | 88 (101) |  | (46) |
|  | UAE | 41 | 81 | all | - | 621 | - | - | 91 | - | (27) |
|  | Brazil | 101 | 173 | all | 234 | - | 399 | 310 | - | - | (44) |
|  | Finland | 802 | 802 | all | - | - | - | 355 | - | - | (45) |
|  | Malaysia | 26 | 103 | all | 651 | - | - | - | 84 | 41 | (26) |

2'-FL, 2'-fucosyllactose; 3-FL, 3-fucosyllactose; LNT, Lacto-N-tetraose; LNnT, Lacto-N-neotetraose; 3'-SL, 3'-sialyllactose; 6'-SL, 6'-sialyllactose.
